# Needs, Perceptions and Education in Sarcoidosis: A Live Interactive Survey of Patients and Partners

**DOI:** 10.1007/s00408-018-0144-4

**Published:** 2018-08-07

**Authors:** C. C. Moor, M. J. G. van Manen, P. M. van Hagen, J. R. Miedema, L. M. van den Toorn, Y. Gür-Demirel, A. P. C. Berendse, J. A. M. van Laar, M. S. Wijsenbeek

**Affiliations:** 1 Department of Respiratory Medicine, Erasmus University Medical Center, s-Gravendijkwal 230, Rotterdam, 3015 CE The Netherlands; 2 Departments of Immunology and Internal Medicine, Erasmus University Medical Center, Rotterdam, The Netherlands; 3 Dutch Sarcoidosis Patient Organisation (Sarcoidose.nl), Alkmaar, The Netherlands

## Abstract

**Objectives:**

Sarcoidosis is a chronic, multisystem disease with often a major impact on quality of life. Information on unmet needs of patients and their partners is lacking. We assessed needs and perceptions of sarcoidosis patients and their partners.

**Methods:**

During patient information meetings in 2015 and 2017 in the Erasmus University Medical Center, we interviewed patients and partners using interactive voting boxes. Patients responded anonymously to 17 questions. Answers were projected directly on the screen in the room.

**Results:**

210 patients and 132 partners participated. Sarcoidosis has a subjective significant impact on lives of both patients and partners. The vast majority of patients and partners feel regularly misunderstood because of the general unawareness of sarcoidosis. Many patients and partners experience anxiety. Three-quarters of patients would like to see more attention and support for their psychological problems. Additionally, more supportive care for partners of sarcoidosis patients is warranted. Interactive interviewing was considered educational (91%) and pleasant (84%).

**Discussion:**

This study improves awareness of needs and perceptions of patients with sarcoidosis and their partners. Sarcoidosis leads to anxiety and psychological distress and impairs well-being of patients and their partners. Attention for psychological support, better disease education, and more supportive care for partners is warranted.

## Background

Sarcoidosis is a heterogeneous, granulomatous disorder of unknown cause, most often localized in the lungs and lymphatic system. However, sarcoidosis can affect almost every organ. Therefore, disease presentation and behavior vary and can be unpredictable [1, 2]. Sarcoidosis often occurs in relatively young adults, between 20 and 50 years of age [2, 3]. Quality of life (QoL) is often impaired due to burden of symptoms such as fatigue, pain, dyspnea, persistent cough, and reduced exercise intolerance. These symptoms can lead to stress, anxiety and depression, and social and physical limitations [4–7]. Side-effects of treatment and complications of disease can also negatively impact QoL [6–8]. Only a few studies aimed to improve QoL in patients with sarcoidosis; these studies were mainly focused on pulmonary rehabilitation [9, 10].

Although it is well-known that sarcoidosis is a disabling disease [7, 11], studies on patients’ needs and preferences in care are lacking. Also, to our knowledge, no currently available studies assessed whether sarcoidosis also influences well-being of partners or other close relatives.

Every year a multidisciplinary sarcoidosis patient meeting takes place in the Erasmus University Medical Center Rotterdam in the Netherlands, aiming to provide up-to-date information and new insights on sarcoidosis to patients and their partners. All patients with confirmed sarcoidosis from the Erasmus University Medical Center are invited to visit these patient meetings. Several medical specialties involved in sarcoidosis care, and the Dutch sarcoidosis patient organization (sarcoidose.nl), organize and attend these meetings.

These meetings allow us to ask patients and partners multiple questions with the use of an interactive voting system. This system enables the attendants to directly see the aggregated results, thereby providing live information about experiences and needs of other patients and partners. A study in pulmonary fibrosis showed that the use of an interactive voting system is considered informative and appreciated by participants, and that it could be an efficient way to inform and educate patients and partners [12].

The aim of this study was to evaluate the needs and perceptions of patients with sarcoidosis and their partners. Moreover, we assessed whether interactive interviewing could be used to enhance education in patients with sarcoidosis.

## Methods

In 2015 and 2017 patients were interviewed during patient information meetings in the Erasmus University Medical Center, one of the two recognized sarcoidosis expert centers in the Netherlands. Patients and partners received voting boxes (TurningPoint 2008; Keepad Interactive, Sydney, Australia) at the start of the information meeting and voted anonymously. Participants were asked permission to use the data before the meeting started. Medical Ethical Committee approval was granted. In accordance with the study of van Manen et al. [12] the term “partners” comprised also other nearest and dearest. Fifteen questions were asked during the meetings in 2015 and 2017. Three questions, about organ involvement and the value of interactive voting, were added in 2017. Literature search, input from patients, physicians, and specialist nurses were used to compose the questions. Moreover, the validated Generalized Anxiety Disorder-Single Item (GAD-SI) was administered [13]. Questions were shown on a big screen and read out loud by one of the speakers. Subsequently, a 10 s countdown was projected on the screen to provide enough time for participants to vote. Afterwards, answers of participants were shown on the screen and directly discussed with the audience. Data were exported and analyzed in Microsoft Excel 2010 afterwards. All data are presented as % (*n*).

## Results

A total of 210 sarcoidosis patients and 132 partners participated in the interactive voting during the two information meetings. Of the 342 participants, 40 people attended both meetings.

In 2017, patients (*n* = 104) were asked to report which organs were involved in their sarcoidosis; 47% of patients reported multi-organ involvement, 34% reported only pulmonary involvement, and a small minority of patients reported respectively only neurological involvement (7%), eye involvement (6%), joint/muscle involvement (3%), cardiac involvement (3%), and skin involvement (1%).

**Fig. 1:**
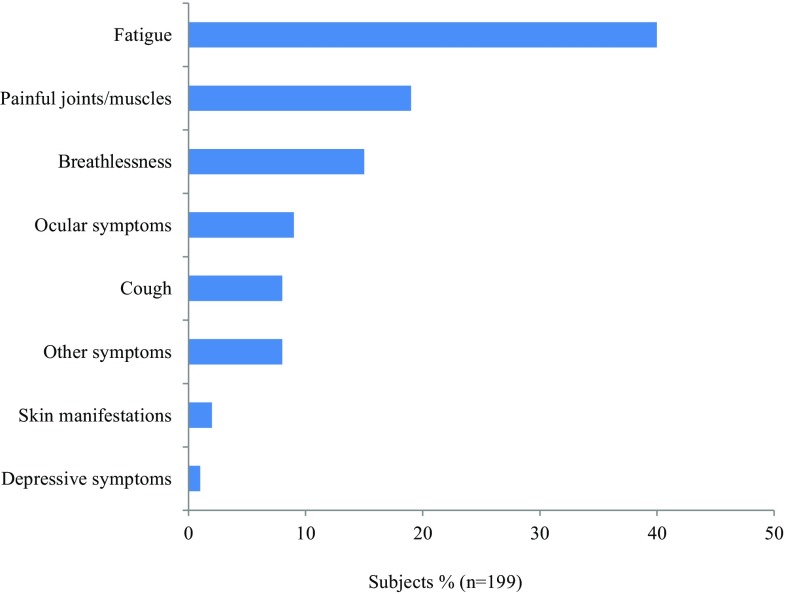
Patients’ response to the question “What symptom of sarcoidosis affects you the most?”

The symptoms that affected sarcoidosis patients most were fatigue, painful joints and/or muscles, and breathlessness. Furthermore, cough, skin manifestations, ocular complaints, and depressive symptoms were reported by a minority of patients as their most disabling symptom (Fig. 1).

**Fig. 2:**
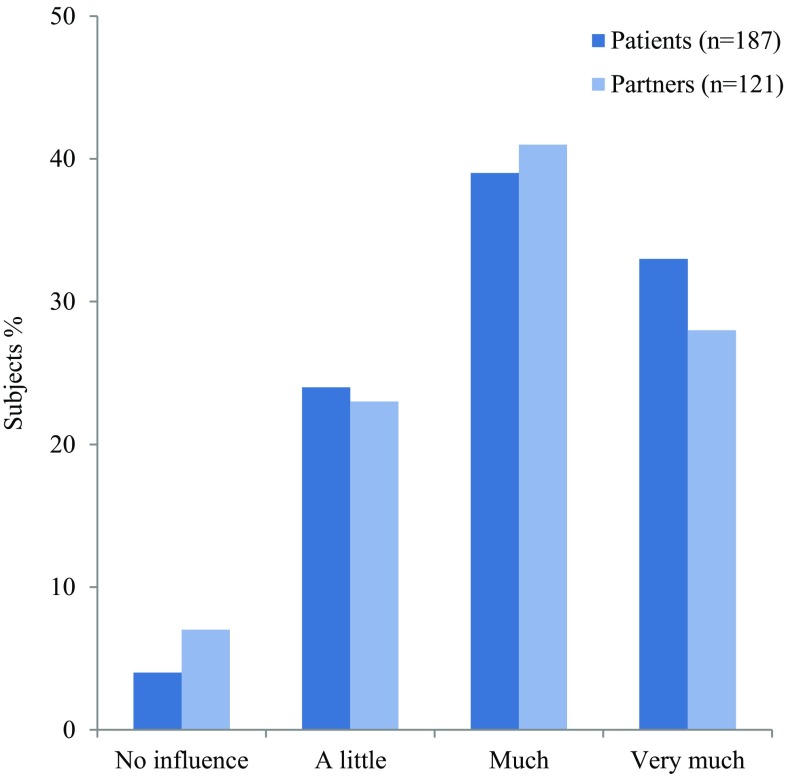
Subject responses to the questions “What is the influence of sarcoidosis on your life at this moment?” and “What is the influence of having a partner with sarcoidosis on your life at this moment?”

Sarcoidosis had a huge impact on the lives of the majority of patients and their partners in a similar manner; almost three-fourth of patients reported (very) much influence on their daily life (Fig. 2).

**Fig. 3:**
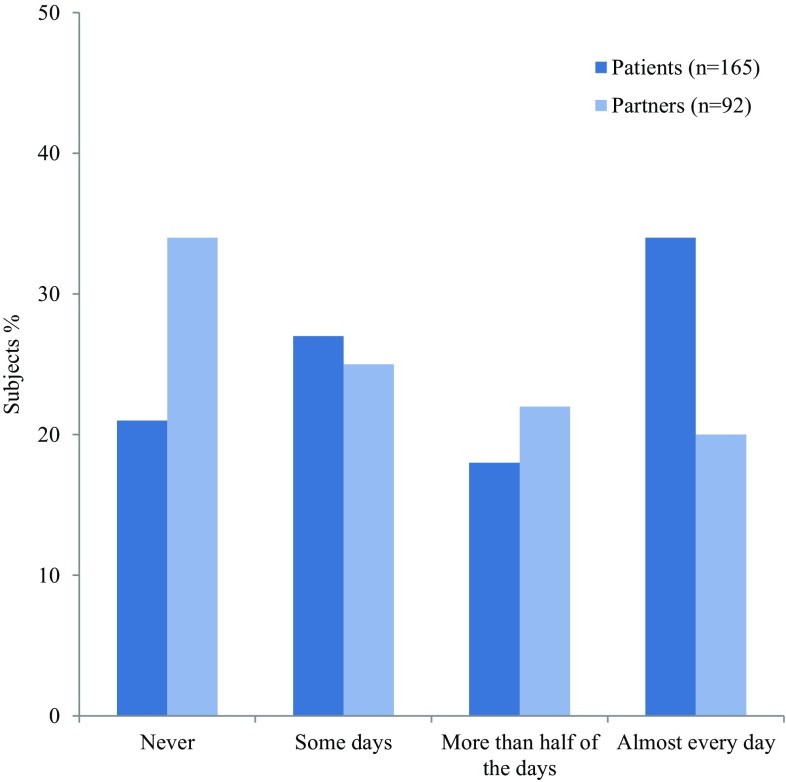
Experience of anxiety of both patients and partners based on the Generalized Anxiety Disorder-single item questionnaire [13] “How often in the past 2 weeks did you have trouble relaxing?”

In the vast majority of patients and partners the GAD-SI score was elevated, which indicates high levels of anxiety (Fig. 3). The answers “more than half of the days” (19%, *n* = 50) and “almost every day” (29%, *n* = 74) based on the GAD-SI questionnaire are considered suggestive of having a generalized anxiety disorder (GAD) [13]. Almost three-quarters of patients (74%, *n* = 132) would like to see more attention and support for their psychological problems. One-third of patients (33%, *n* = 59) stated that they missed psychological support in standard care, whereas 41% (*n* = 73) of patients would only like to receive psychological care when they specifically ask for it. A minority of patients reported no psychological issues (18%, *n* = 32).

**Fig. 4:**
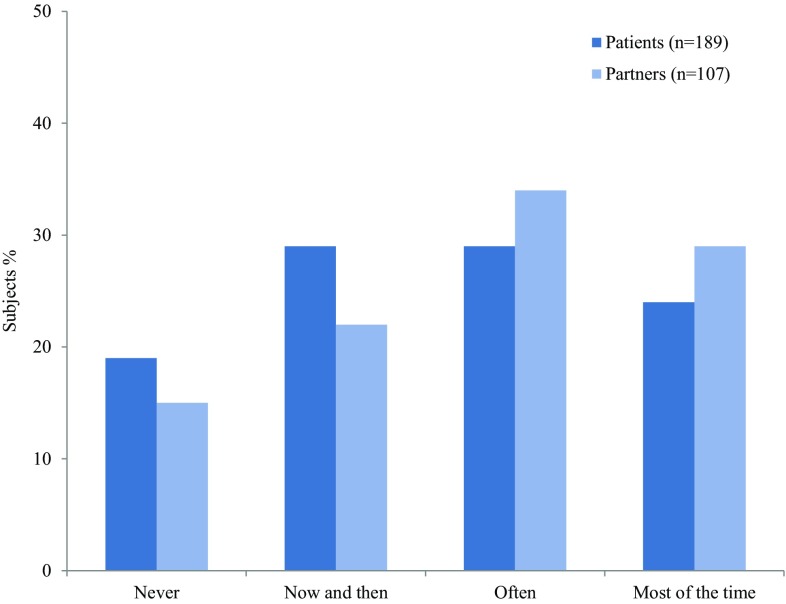
Participants’ responses to the question “How often do you experience misunderstanding because people do not know what sarcoidosis is?”

In addition, many participants experienced some degree of misunderstanding, because of the general unawareness of sarcoidosis. Figure 4 shows that partners seem to experience even more misunderstanding than patients.

The main needs in care of patients with sarcoidosis were easy access to an expert center for sarcoidosis (36%, *n* = 67) and receiving adequate information about the disease (41%, *n* = 78). Additionally, the importance of practical and emotional support, and contact with peers were mentioned by patients. Also, 41% (*n* = 53) of partners thought that there should be more support for the partners of sarcoidosis patients.

Furthermore, we asked patients questions about their opinion on eHealth. The majority of participants (70%, *n* = 132) would like to keep track of their data and symptoms on the internet. Almost all patients (92%, *n* = 170) would be willing to measure lung function at home to optimize treatment.

**Fig. 5:**
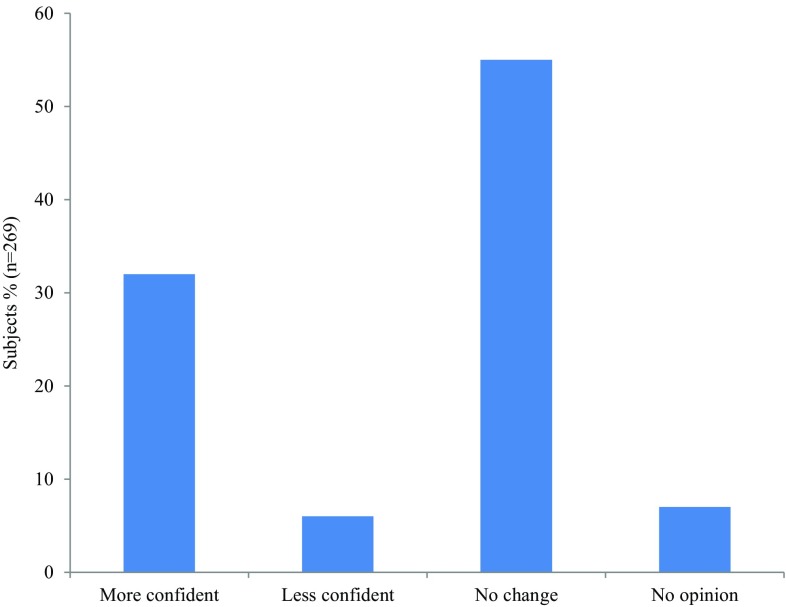
Feelings of patients and partners at the end of the meeting

Most patients and partners rated the information meeting as very useful (86%, *n* = 237). About one-third of participants felt more confident after the meeting (Fig. 5). In 2017, 84% (*n* = 128) of participants appreciated seeing the answers of other participants immediately after each question, and 91% (*n* = 136) considered the interactive interviewing educational.

## Discussion

This study shows the major psychological and social impact of sarcoidosis, not only on patients but also on their partners. Education and psychological support are reported as important unmet needs. Furthermore, the study shows that patients and partners appreciate the interactive voting system and consider it informative. Interactive voting might be a suitable method to facilitate discussion with patients and to educate and support them at the same time.

### The Patient Voice in Care

The importance of patient participation and shared decision making in healthcare has been increasingly acknowledged during the last years [14]. Although studies on patient participation in sarcoidosis are scarce, it is appreciated that engaging patients in care could lead to better clinical outcomes and treatment adherence, especially because sarcoidosis is such a multidimensional disease [11]. Recently, the Netherlands institute for health services research showed that 75% of sarcoidosis patients considers shared decision making important [15]. However, to allow for shared decision making, patients must be well informed and physicians should have better insights into patients’ perception of disease and preferences in care. The data of this study can be used to better address the needs of patients with sarcoidosis and their partners, and to improve daily care.

### Fatigue

Most patients in our study report fatigue as the symptom which affects them most. This is in accordance with previous studies, in which fatigue was the most burdensome and frequent symptom in sarcoidosis [16, 17], with a negative impact on QoL [8, 11, 18]. Fatigue often persists, even if sarcoidosis is treated well and no other sarcoidosis disease activity can be found [11]. A few small studies showed effect of neurostimulants or pulmonary rehabilitation on fatigue [9, 10, 19, 20], however, larger randomized trials with specific fatigue interventions are lacking. Design of such trials is complicated, because sarcoidosis-associated fatigue is a multidimensional, complex problem with unknown etiology [17, 21]. Besides, fatigue in sarcoidosis is related to depressive symptoms and sleep disturbance [17, 18, 22, 23]. As also illustrated by the results of this study, fatigue remains a major problem for patients. A multidisciplinary approach towards sarcoidosis-associated fatigue is needed and should be subject for future research.

### Influence of Sarcoidosis on Daily Life

Our study shows that sarcoidosis has a major influence on patients’ daily lives. It is well established that sarcoidosis has a huge impact on patients; health status and QoL are lower than in the normal population [4, 7, 24]. This study is the first to show that sarcoidosis also has a major impact on the daily lives of partners of sarcoidosis patients.

Many patients feel that they are not taken serious by relatives and friends. The reason for this might be that sarcoidosis is often not visible [11]. In our study, many patients and partners experience misunderstanding because of the general unawareness of sarcoidosis in society. Patients often have non-specific symptoms, such as fatigue, depression, reduced exercise capacity, or pain, which are difficult to quantify. This could contribute to incomprehension of the impact of sarcoidosis, and lead to reduced labor force participation, social isolation, and disturbed relationships [4, 11, 25]. Improving general awareness about sarcoidosis and acknowledging the impact of sarcoidosis on many aspects of life, could possibly help sarcoidosis patients and partners to feel better understood.

### Psychological Problems

Anxiety, stress, and depressive symptoms are common problems in sarcoidosis. In our study, the vast majority of patients experience some level of anxiety [13]. In literature, prevalence of depressive symptoms in sarcoidosis ranges from 27 to 66% and prevalence of anxiety ranges from 5 to 32% [5, 6, 23, 26, 27]. Severe disease, multi-organ involvement and dyspnea are associated with more depressive symptoms [26, 27]. However, the design of our study does not permit looking at such a correlation. Patients’ perception of disease, independent of disease status, might lead to anxiety and depression [27]. Disease chronicity, unpredictable course, and uncertain future perspectives can also impair emotional well-being [23]. In daily practice, these aspects are often neglected, and more recognition and tailored interventions should be stimulated. Examples of possible interventions include cognitive behavioral therapy or psychological counseling [11, 24].

### Anxiety in Partners

Strikingly, not only patients but also two-third of partners experience anxiety, 20% of them almost every day. In other chronic diseases, such as cancer, dementia, and rheumatoid arthritis, it has been acknowledged that many partners encounter psychological distress and have a decreased QoL [28]. Some studies showed that caregivers are even more distressed than patients, and that depression of patients was significantly associated with depression of their partners [28–30]. Partners of sarcoidosis patients in our study report more anxiety and misunderstanding than partners of idiopathic pulmonary fibrosis (IPF) patients as reported previously [12]. This is remarkable when considering the progressive nature of disease and associated severely reduced life expectancy in IPF.

Currently, there are different questionnaires available which evaluate well-being, anxiety, and depressive symptoms of (informal) caregivers [31, 32]. In the light of the results of our study, it should be considered to incorporate such questionnaires in future care and studies, to gain more insight in QoL of partners of patients with sarcoidosis, and its effect on well-being of the patient.

### Preferences in Care

Many partners of sarcoidosis patients in our study think that partners should receive more care. This is in line with studies assessing needs in partners of patients with other chronic illnesses [28, 30]. One of the options mentioned in literature is to invite partners more actively for outpatient clinic visits, encouraging them to ask questions or express concerns, and involve partners in decision making [28].

Clinicians tend to have more attention for physical parameters than psychological issues in patients with sarcoidosis [23]. This is in accordance with findings from our study, in which the majority of patients would appreciate more attention for psychological care. Furthermore, patients report the importance of access to an expert center, practical support, and contact with other patients. Patient organizations can also play an important role in facilitating contact with expert centers, information, and peer support.

### Home Monitoring

Use of eHealth technologies has been increasing in recent years, and eHealth studies show promising results for improving quality of care [33]. The majority of patients in our study wish to keep track of their symptoms and manage their personal data at home using an internet tool. Furthermore, most patients in the current study are also willing to measure lung function at home. These are encouraging results for future care and trials, because a recent study in patients with newly diagnosed sarcoidosis showed that home spirometry was feasible and allowed for early detection of steroid treatment effects [34]. Home monitoring of lung function, symptoms, and side-effects, can help physicians to enhance individually tailored treatment by minimizing side-effects, maximizing effects and engaging patients in care [34].

### Education

One of the main unmet needs in sarcoidosis care revealed in this study is the need for more information about the disease. In a recent government survey about chronic lung diseases, more than half of Dutch sarcoidosis patients reported that they cannot find sufficient information about their disease and its prospects [15]. This is one of the reasons that sarcoidosis patient information meetings are organized every year. However, literature about the best method to provide information to patients with sarcoidosis is scarce. Drent and colleagues [11] state that the complex etiology of sarcoidosis and its variability make it complicated to provide adequate information, and that “affective communication” probably makes it easier for patients to remember medical information. In the current study, one-third of patients felt more secure after the information meeting and the vast majority of patients appreciated live interactive interviewing, showing that this may be a promising method to enhance education of patients and partners.

### Limitations

This study has of course limitations. Because of the interactive voting system, no specific patient characteristics are available, such as age, gender, and disease duration. Organ involvement was self-reported by patients and could not be verified. Furthermore, this was a single center study. Despite these limitations, we believe that the results are relevant for a broader group of sarcoidosis patients. We invited all patients with confirmed sarcoidosis of the Erasmus Medical Center, including patients with a wide spectrum of disease manifestations and severity. A small minority of patients attended both the 2015 and 2017 meeting. However, because the total group of participants is large, the estimated effect of overlap in data is only small. Furthermore, not all patients answered all questions. Reasons for not answering could be preference not to answer certain questions or being too late to respond. Therefore, we expressed all results as n (%), since the response rate might differ per question.

## Conclusion

This study improves awareness of needs and perceptions of both patients and their partners in sarcoidosis. Sarcoidosis not only leads to anxiety and psychological distress and impaired well-being in patients, but also in their partners. Therefore, attention for psychological support, better disease education, and more care for partners is warranted. Besides the ongoing need for improvement of disease modifying agents, future research should also focus on patient-centered programs to relieve distress and improve QoL for both sarcoidosis patients and their partners.

## Data Availability

The datasets used and/or analysed during the current study are available from the corresponding author on reasonable request.
